# Molecular subgrouping of medulloblastoma in pediatric population using the NanoString assay and comparison with immunohistochemistry methods

**DOI:** 10.1186/s12885-022-10328-6

**Published:** 2022-11-28

**Authors:** Joo Whan Kim, Sung-Hye Park, Seung Ah Choi, Seung-Ki Kim, Eun Jung Koh, Jae-Kyung Won, Sun Mo Nam, Ji Hoon Phi

**Affiliations:** 1 Division of Pediatric Neurosurgery, Seoul National University Children’s Hospital, Seoul National University College of Medicine, 101 Daehak-Ro, Jongno-Gu, 03080 Seoul, Republic of Korea; 2 Department of Pathology, Seoul National University Children’s Hospital, Seoul National University College of Medicine, Seoul, Republic of Korea

**Keywords:** Medulloblastoma, Molecular, Subgroup, Method, Immunohistochemistry

## Abstract

**Purpose:**

Molecular subgrouping of medulloblastoma has become important due to its impact on risk group stratification. Immunohistochemistry (IHC) has been widely used but it has innate limitations. The NanoString assay has been proposed as an alternative method. This study aims to present the characteristics of medulloblastoma subgrouped by the NanoString assay and to compare the subgrouping results with the IHC method.

**Methods:**

Pediatric patients with histological diagnosis of medulloblastoma who underwent surgery from 2007 to 2021 were included. Clinical characteristics, pathological findings were reviewed. Molecular subgrouping was performed by IHC and by NanoString nCounter Elements TagSets assay. Test for concordance between two methods was made.

**Results:**

Among a total of 101 patients analyzed, subgrouping using the NanoString assay resulted in 14 (13.8%) WNT, 20 (19.8%) SHH, 18 (17.8%) Group 3, and 39 (38.6%) Group 4 subgroup cases. Survival analysis revealed the following from best to worse prognosis: WNT, Group 4, SHH, and Group 3. In SHH subgroup the large cell/anaplastic histology was present in 30% of cases. Seventy-one cases were analyzed for concordance between NanoString and IHC. Cohen’s kappa value indicated moderate agreement but identification of Groups 3 and 4 with IHC using NPR3 and KCNA1 markers exhibited poor results.

**Conclusions:**

The NanoString assay of Korean medulloblastoma patients revealed a more aggressive clinical course in the SHH subgroup which may be explained by a higher proportion of large cell/anaplastic histology being present in this subgroup. IHC did not distinguish Group 3 or 4 accurately. The NanoString assay may represent a good alternative method for practical use in the clinical field.

**Supplementary Information:**

The online version contains supplementary material available at 10.1186/s12885-022-10328-6.

## Background

Medulloblastoma occupies an important position as it is the most common malignant brain tumor in children [1]. Established as a disease entity in the 1920s by Harvey Cushing and Percival Bailey [2], medulloblastoma remains a single disease with little variability with respect to the age of onset, tumor location, and histology. However, the divergent prognoses in many subgroups of patients necessitated a more detailed classification. Around 2010, several groups reported that medulloblastomas comprise at least 4 distinct molecular subgroups: Wingless signaling-activated (WNT), Sonic-hedgehog signaling-activated (SHH), Group 3, and Group 4, largely based on transcriptome profiles and a few known genetic alterations [3, 4]. Thereafter, molecular subgrouping of medulloblastoma became an important step of risk stratification, reflected in upcoming 2021 World Health Organization classification in which molecular subgrouping emerges as the mainstream [5, 6].

Currently, in the era of next-generation sequencing, the biology of medulloblastoma is being scrutinized with more comprehensive whole-genome, transcriptome, and methylome analyses [7, 8]. However, clinically more succinct and economic methods are required for quick and easy identification of molecular subgroups of brain tumors. Immunohistochemistry (IHC) has been used for the differential diagnosis of brain tumors for a long time. IHC-based methods using representative markers of each medulloblastoma subgroup were considered acceptable and have been used in many institutions where in-house molecular diagnosis is not available [9, 10].

Although the IHC method is used in clinical setting, it has innate limitations. The NanoString nCounter assay has been proposed as an alternative method to overcome the limitations of IHC. In recently published studies, the NanoString assay exhibits more sensitive and accurate identification in the molecular subgrouping of medulloblastoma than the IHC method [10, 11]. In addition, affordable cost and turn around time, reproducibility of results also promotes the use of NanoString assay [11, 12].

In Korea, most large centers have utilized IHC methods for the diagnosis and subgrouping of medulloblastoma. However, to our knowledge, the distribution and clinical features of the four molecular subgroups have not been reported for Korean pediatric patients in any literature [9, 13]. There is evidence that racial and ethnic differences exist in the incidence and characteristics of 4 molecular subgroups of medulloblastoma [14, 15]. In this study, the proportions and characteristics of molecular subgroups using NanoString assay are presented. The feasibility and utility of the NanoString method in comparison with IHC for clinical practice are also examined.

## Methods

### Patients

Included patients were those who underwent surgery at Seoul National University Children’s Hospital between January 2007 and February 2021 and were pathologically confirmed as having medulloblastoma. Clinical characteristics, pathological findings, and IHC results were reviewed. For comparison between the NanoString assay and IHC method, patients were excluded if they had insufficient data to determine their subgroup using IHC methods. All patients were treated with a standardized treatment protocol, including upfront maximal surgical resection of tumors followed by radiation of the tumor bed with the craniospinal axis (over the ages of three) and chemotherapy. High-risk patients received high-dose radiation and high-dose chemotherapy with autologous peripheral blood stem cell transplantation [16]. This study protocol was approved by the local Institutional Review Board (IRB No. 1807–069-958) and was conducted according to the Helsinki Declaration.

### NanoString nCounter elements analysis

To identify the molecular subgroups of medulloblastoma, NanoString nCounter Elements Taqsets analysis (NanoString Technologies, Seattle, WA) was performed as previously described [17]. CodeSet was designed with a total of 25 genes, including 3 housekeeping genes (Supplementary Table 1). The probe set for each gene in CodeSet was synthesized by IDT (Integrated DNA Technologies, Inc.) for NanoString nCounter Elements analysis. Total RNA was isolated from snap-frozen tumor tissues (*N* = 111) using RNeasy kits (Qiagen, Hilden, Germany) and RNA integrity was verified using an Agilent 2100 Bioanalyzer (Agilent Technologies, Santa Clara, CA). The algorithm for group assignment was provided by Dr. M. Taylor (Toronto University, Canada) [12]. All procedures including sample preparation, hybridization, detection, normalization, scanning and analysis were accomplished according to the NanoString Technologies instruction (NanoString Technologies, Seattle, WA). In this study, only snap freezing samples that passed the sample quality requirements according to the strict criteria for NanoString element assay guidelines were used, and samples presented as failures due to RNA quality were excluded from this study. Samples older than 8 years (*N* = 9), were considered old samples according to suggested cut-off [12]. Reliability of results using theses samples were validated with our own previous NanoString analysis results [17].

### Histopathology, IHC, fluorescence in situ hybridization and tumor gene panel sequencing

**Fig. 1:**
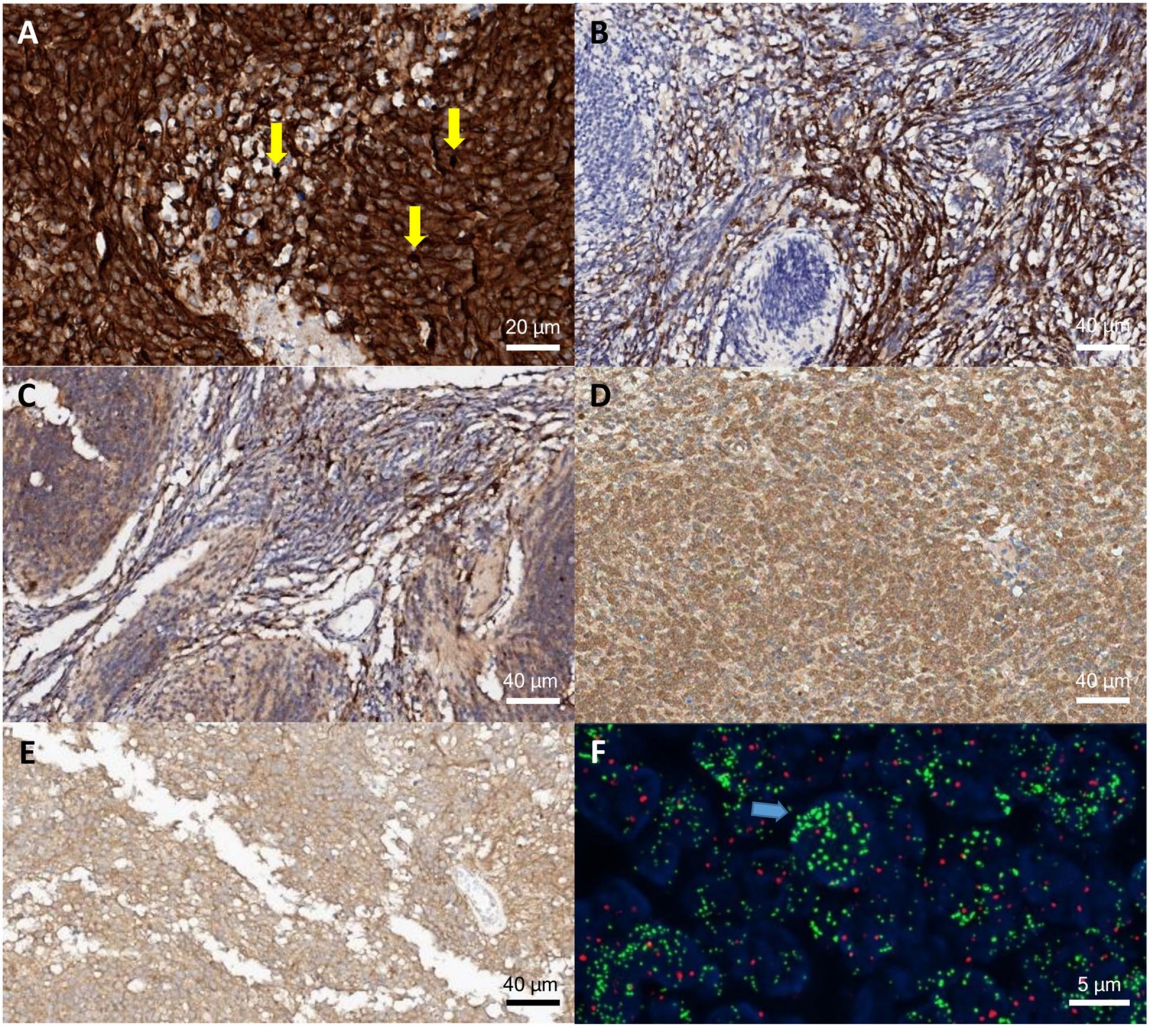
IHC expression and FISH results. Nuclear beta-catenin expression (**A**, yellow arrow), GAB1 (**B**) and SFRP1 (**C**) expression was seen. NPR3 (**D**) and KCNA1 (**E**) was seen for Group 3 and 4. FISH shows *MYCN* amplification (**F**), specimens containing either more than 10 signals or innumerable tight clusters of signals (F, blue arrow) in more than 10% of tumor cells were considered of amplification

Histology was classified into 4 subtypes, including classic, medulloblastoma with extensive nodularity, desmoplastic/nodular, and large cell/anaplastic, according to the revised 4th World Health Organization classification of CNS tumors (2016) by the Department of Pathology of our institute. The IHC staining protocol and antibodies used were the same as those in a previous report from our institute [9]. IHC staining was performed according to the protocol of the manufacturer, based on a biotin-free polymer detection system. The antibodies used were beta-catenin (1:200; BD Biosciences, Franklin Lakes, NJ), DKK1 (1:200; 2A5; Abnova, Taipei, Taiwan), YAP (1:50; sc-101199; Santa Cruz Biotechnology, Santa Cruz, CA), filamin A (1:300; PM6/317; Fitzgerald, Acton, MA), GAB1 (1:50; Abcam, Cambridge, MA), SFRP1 (1:300; Abcam), NPR3 (1:800; Abcam), and KCNA1 (1:500; Abcam). Positive immunolabeling was defined as uniform intense labeling in nuclear and cytoplasm (beta-catenin, filamin A), or cytoplasm and cytoplasmic membrane (DKK1, YAP, GAB1, SFRP1, NPR3, KCNA1) in more than 10% of the tumor area (Fig. 1a-e) [9].

Diagnostic criteria of molecular subgrouping using the IHC method were established based on the literature [18]. The presence of beta-catenin labeling in the tumor cell nuclei was considered a positive result and determined to be a WNT subgroup. If nuclear beta-catenin stain is weak, positive DKK with YAP or filamin A positivity was considered to be a WNT subgroup. Positive GAB1 or SFRP1 with negative beta-catenin were determinants of the SHH subgroup. NPR3 positivity without WNT or SHH markers was classified as Group 3. KCNA1 positivity without WNT or SHH markers was classified as Group 4. All markers negative or both NPR3 and KCNA1 positivity without WNT or SHH markers were classified as unclassifiable. Fluorescence in situ hybridization (FISH) for *MYC* and *MYCN* amplification was performed using the same protocol described in preliminary report [9]. FISH was performed on unstained FFPE array slides as described using commercially available digoxigenin-labeled cosmid probes for *MYC* (8q24.12-q24.13; orange; Vysis, Downers Grove, IL), reference probe CEP8 (8p11.1-q11.1; green; Vysis), *MYCN* (2p24; green; Vysis), reference probe CEP2 (2p11.1-q11.1; orange; Vysis). Slides were deparaffinized and treated with proteinase K, then denatured and treated with prediluted probes and hybridized overnight. Values for each signal and the ratios of green/red signals were reported in at least 100 nonoverlapping nuclei per specimen. Specimens containing either more than 10 signals or innumerable tight clusters of signals in more than 10% of tumor cells were considered *MYC* or *MYCN* amplification (Fig. 1f) [9]. To analyze important genetic alterations, we conducted tumor gene panel screening for known driver mutations/fusions/copy number alterations frequently found in brain tumors since June 2018 and it was performed on 23 cases included in this study. The gene panel sequencing consists of comprehensive genomic alterations and representative genes for medulloblastoma are *CTNNB1, GLI1, MYCN, PTCH1, PTCH2, PTEN, SUFU, TP53, YAP1* [19].

### Statistical analysis

The SPSS software version 19 (SPSS Inc., Chicago, USA) was used for statistical analysis. To evaluate statistical significance between categorical variables, the chi-square test for independence was used. Additionally, Cohen’s kappa coefficient was calculated to determine the agreement between two diagnostic methods. The R (version 4.1.1) software was used for graphics and statistical analysis. Kaplan–Meier and log-rank test was performed using *survminer* package (https://cran.r-project.org/web/packages/survminer/index.html, last published March 9, 2021). Alluvial plot was made using *ggalluvial* package (https://cran.r-project.org/web/packages/ggalluvial/index.html, last published December 5, 2020). A *p*-value less than 0.05 was considered statistically significant.

## Results

### Patient characteristics

**Fig. 2:**
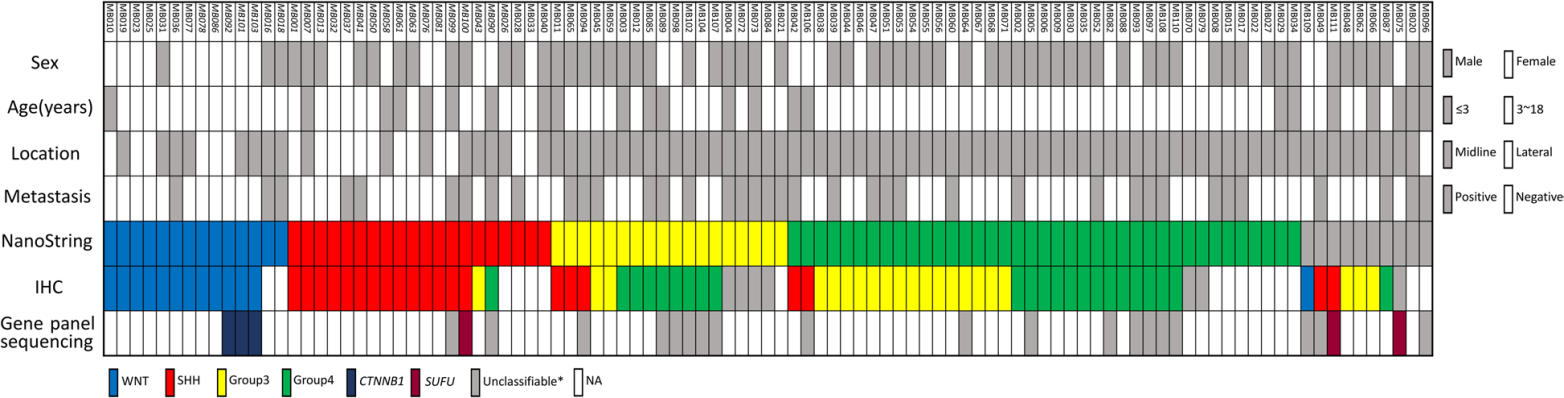
A summary of molecular subgrouping by the NanoString assay and IHC of 101 patients with gene panel sequencing and clinical characteristics. IHC, Immunohistochemistry; WNT, Wingless signaling-activated; SHH, Sonic-hedgehog signaling-activated; NA, Not available. * ‘Unclassifiable’ in gene panel sequencing indicates absence of subgroup-defining genetic alterations such as mutations in *CTNNB1* or *SUFU* gene

A NanoString assay was performed in 111 medulloblastoma tissues. Among them, 10 tissues were recurrent tumors with corresponding initial tumor tissues in the cohort. Their clinical features are not duplicated in the patient characteristic table. Therefore, a total of 101 medulloblastoma patients with molecular subgrouping with the NanoString assay were analyzed (Supplementary Table 2). The mean age at diagnosis was 7.7 years. Male predominance was observed with 63 (62.4%) males and 38 (37.6%) females. Twenty-three (22.7%) patients were diagnosed younger than three years of age and 78 (77.2%) patients were between three to 18 years of age. Metastasis at the time of diagnosis was seen in 38 (37.6%) patients. Extent of surgery was gross total resection in 49 (48.5%) cases, near total resection in 42 (41.6%) cases, and subtotal resection in 10 (9.9%) cases. Histologic features of the samples revealed 61 (60.4%) classic type, 17 (16.8%) medulloblastoma with extensive nodularity, 9 (8.9%) desmoplastic/nodular type, and 14 (13.9%) large cell/anaplastic type. Subgrouping using the IHC method resulted in 13 (12.9%) WNT subgroup cases, 22 (21.8%) SHH subgroup cases, 21 (20.8%) Group 3, and 23 (22.8%) Group 4. Seven (6.9%) cases were unclassifiable and 16 (15.8%) cases had insufficient data to determine molecular subgroup. Subgrouping using the NanoString assay revealed 14 (13.9%) WNT subgroup cases, 20 (19.8%) SHH subgroup cases, 18 (17.8%) Group 3, and 39 (38.6%) Group 4 with ten (9.9%) cases being unclassifiable. A summary of all 101 cases with their clinical characteristics and molecular subgrouping using the NanoString assay and IHC is shown in Fig. 2.

### Molecular subgrouping by the NanoString assay

**Fig. 3:**
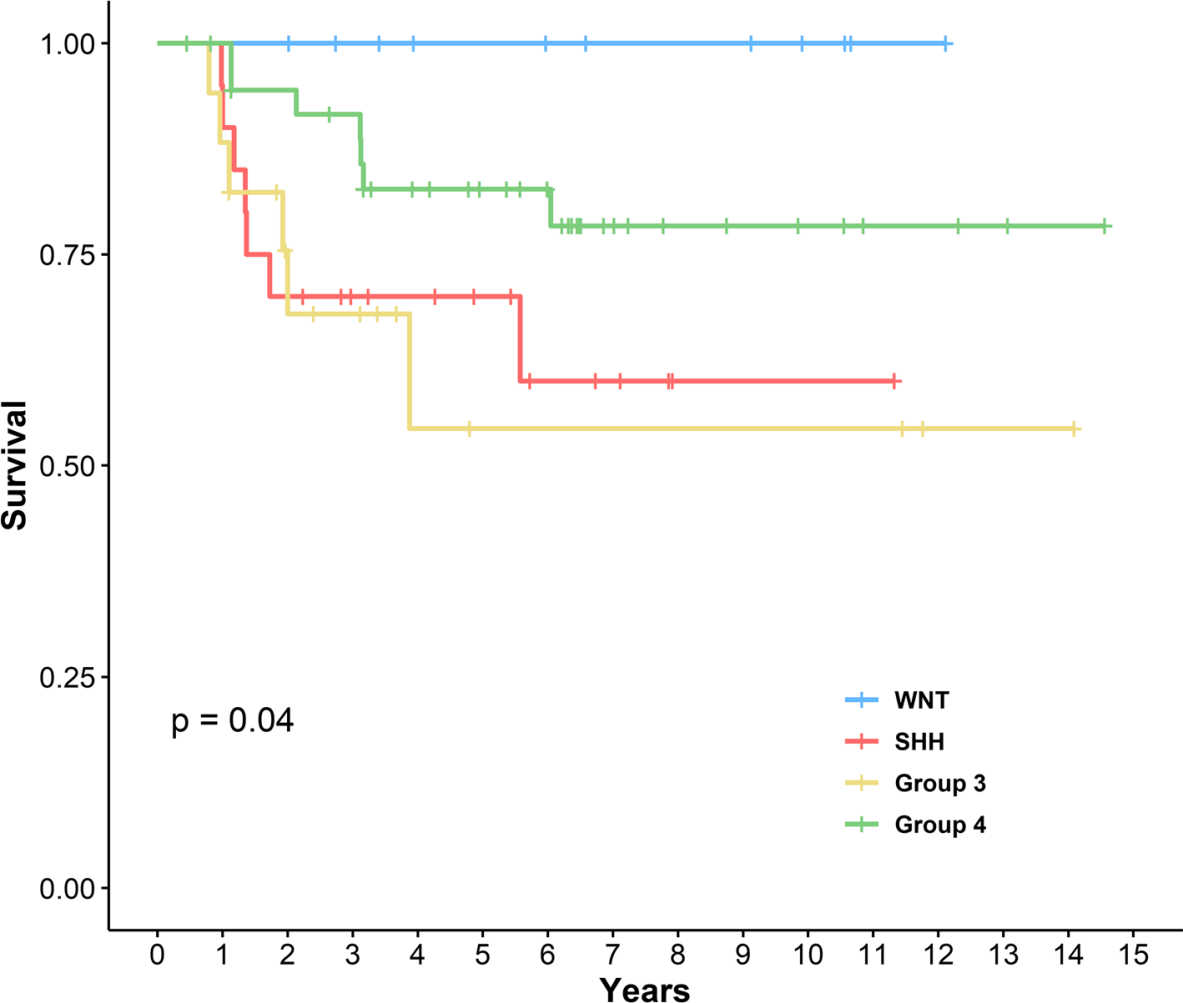
Kaplan–Meier plot for overall survival of molecular subgroups. WNT, Wingless signaling-activated; SHH, Sonic-hedgehog signaling-activated. *P* value was calculated using the Log-rank test

**Fig. 4:**
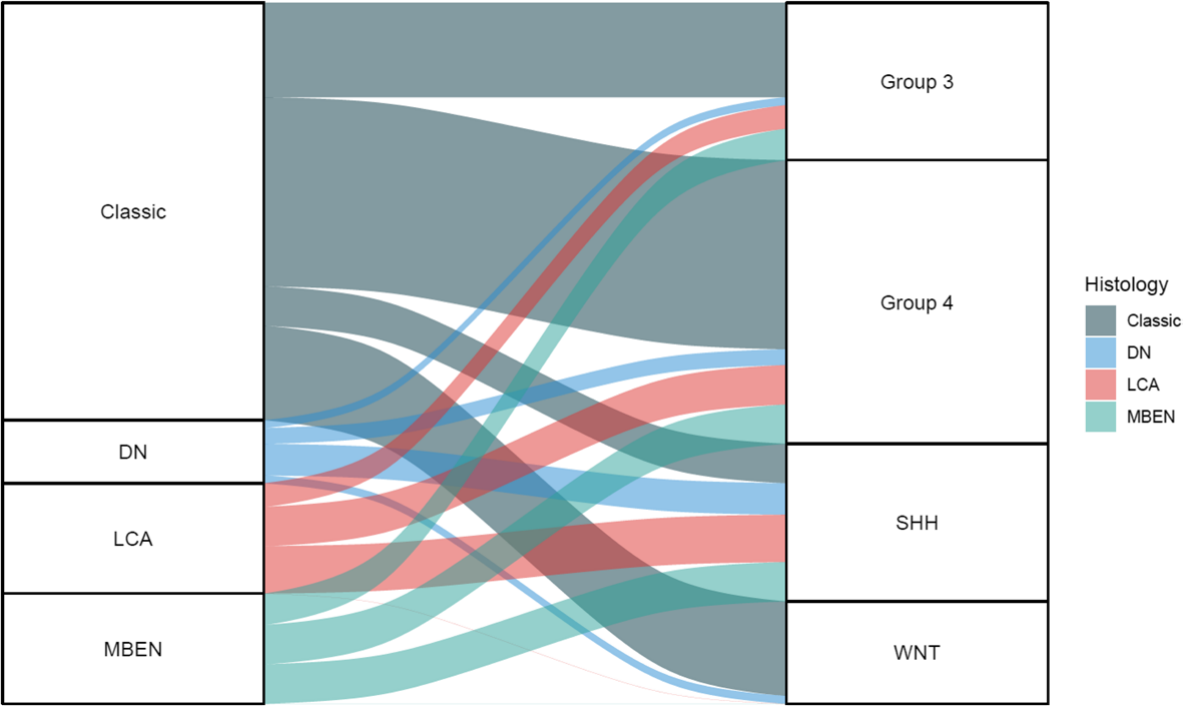
Alluvial plot for proportion of histology in molecular subgroups. DN, Desmoplastic/nodular; LCA, Large cell/anaplastic; MBEN, Medulloblastoma with extensive nodularity; SHH, Sonic-hedgehog signaling-activated; WNT, Wingless signaling-activated

Clinical characteristics of the subgroup by NanoString assay are reviewed (Table 1). A gender difference was observed between subgroups with female predominance in the WNT subgroup (M:F = 3:11) and male predominance in Group 3 (M:F = 14:4) and Group 4 (M:F = 28:11). Age and metastasis at diagnosis did not exhibit a difference among the groups. The location of the tumor displayed a significant difference with subgroups. The cerebellar peduncular location was only seen in the WNT subgroup and the cerebellar hemispheric location was only seen in the SHH subgroup. Regarding prognosis, the 4 subgroups clearly exhibited differences in overall survival. Kaplan–Meier curves showed the following from best to worse prognosis: WNT, Group 4, SHH, and Group 3 (Fig. 3). The histologic features of WNT, Group 3, and Group 4 showed the classic type accounting for the majority of cases. However, in the SHH subgroup histological features were evenly distributed and the large cell/anaplastic type accounted for up to 30% of cases (Fig. 4). There were 10 unclassifiable patients as assessed using the NanoString assay. According to the IHC criteria these 10 patients included one WNT, two SHH, three Group 3, and one Group 4 (Fig. 2).

**Table 1:** Characteristics of subgroup (by the NanoString assay)

Clinical Features	Number of Cases (%) or value				
	WNT (*N* = 14)	SHH (*N* = 20)	Group 3 (*N* = 18)	Group 4 (*N* = 39)	*P* value
Gender					0.003
M	3 (21.4%)	11 (55.0%)	14 (77.8%)	28 (71.8%)	
F	11 (78.6%)	9 (45.0%)	4 (22.2%)	11 (28.2%)	
Age (years)					0.007
≤ 3	1 (7.1%)	7 (35.0%)	7 (38.9%)	3 (7.7%)	
3–18	13 (92.9%)	13 (65.0%)	11 (61.1%)	36 (92.3%)	
Metastasis at diagnosis					0.096
Negative	11 (78.6%)	14 (70.0%)	7 (38.9%)	25 (64.1%)	
Positive	3 (21.4%)	6 (30.0%)	11 (61.1%)	14 (35.9%)	
Tumor location					< 0.001
Midline	8 (57.1%)	8 (40.0%)	18 (100.0%)	39 (100.0%)	
Hemispheric	0 (0.0%)	12 (60.0%)	0 (0.0%)	0 (0.0%)	
Peduncle	6 (42.9%)	0 (0.0%)	0 (0.0%)	0 (0.0%)	
Histology					0.018
Classic	13 (92.9%)	5 (25.0%)	10 (55.6%)	27 (69.2%)	
MBEN	0 (0.0%)	5 (25.0%)	4 (22.2%)	5 (12.8%)	
DN	1 (7.1%)	4 (20.0%)	1 (5.6%)	2 (5.1%)	
LCA	0 (0.0%)	6 (30.0%)	3 (16.7%)	5 (12.8%)	

*Abbreviations*: *WNT* Wingless signaling-activated, *SHH* Sonic-hedgehog signaling-activated, *MBEN* Medulloblastoma with extensive nodularity, *DN* Desmoplastic/nodular, *LCA* Large cell/anaplastic

*P* values were calculated using the the chi-square test for independence

### Comparison of the NanoString assay vs. IHC

Of 101 patients, 15 who had insufficient IHC data before 2012 were excluded, and 86 were included in the comparative analysis. With the exclusion of unclassifiable cases using either NanoString or IHC, 71 cases were analyzed for concordance. From a total of 71 patients, agreement between the NanoString and IHC subgrouping results was observed in 41 patients (57.7%). Cohen’s kappa value (k) was 0.424 indicating moderate agreement between the two methods (Table 2).

**Table 2:** Comparison of the NanoString assay vs. IHC

	NanoString				Number of cases
IHC	WNT	SHH	Group 3	Group 4	
WNT	12	0	0	0	12
SHH	0	14	3	2	19
Group 3	0	1	2	15	18
Group 4	0	1	8	13	22
Number of cases	12	16	13	30	71

*Abbreviations*: *WNT* Wingless signaling-activated, *SHH* Sonic-hedgehog signaling-activated

Based on the NanoString analysis, the WNT group was consistent with IHC in all 12 cases. In the SHH group (*N* = 16), 14 cases showed agreement while two cases did not. Identification of Group 3 and Group 4 with IHC using NPR3 and KCNA1 markers showed poor results. Considering NanoString as a baseline study, IHC misidentified 23 of 38 Groups 3 and 4 cases with a negative Cohen’s kappa value which indicates no agreement between the two methods. There were seven unclassifiable cases as assessed by IHC. The NanoString results for those cases were four Group 3, two Group 4, and one unclassifiable (Fig. 2).

### Analysis of MYC/MYCN amplification and TP53 mutation

**Fig. 5:**
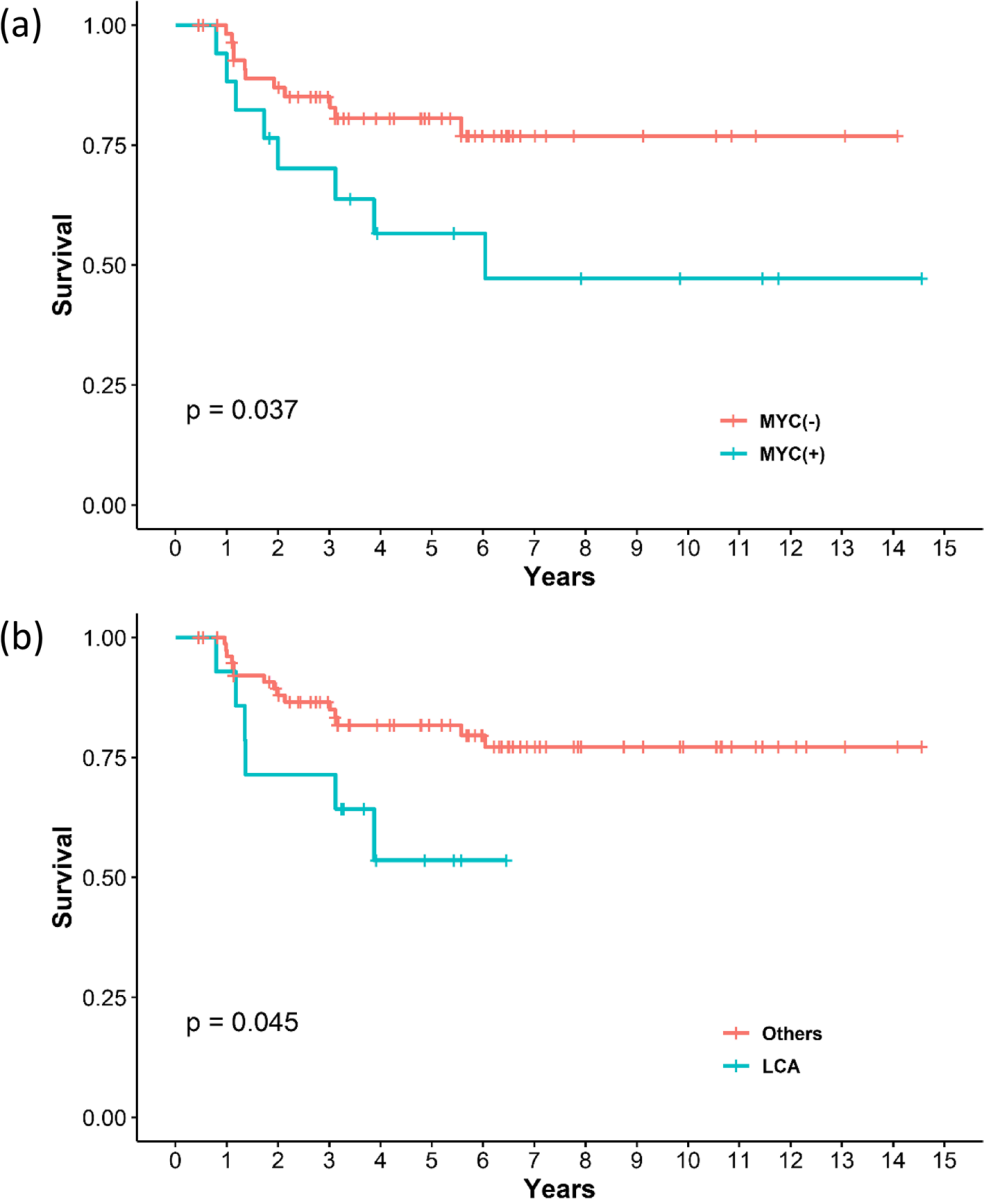
Kaplan–Meier plot for overall survival of (**a**) *MYC/MYCN* amplification and (**b**) Large cell/anaplastic histology. LCA, Large cell/anaplastic. *P* values were calculated using the Log-rank test

FISH for *MYCN* and *MYC* amplification was performed in 69 (81.1%) and 49 (57.6%) respectively out of 81 patients. *MYC*/*MYCN* amplification was observed in 12 (14.8%) patients. Twelve patients consisted of five SHH, four Group 3, and three Group 4 patients. The presence of *MYC/MYCN* amplification conveyed a significant difference in overall survival (Fig. 5a). *MYC/MYCN* amplification showed significant difference between large cell/anaplastic histology and other histologic subtypes, Fisher’s exact test showing *p* value of 0.027.

The presence of *TP53* mutations was analyzed in 25 cases using tumor gene panel sequencing as described above. Before conducting the gene panel study, IHC of p53 was performed in 51 patients. Among them, overexpression of p53 was observed in eight cases in the SHH subgroup, and gene panel sequencing was performed in three cases in the SHH subgroup. None of these three cases with gene panel sequencing exhibited *TP53* mutation; nevertheless, two of eight p53 IHC cases displayed diffuse overexpression of p53 in over 90% of cells stained. The presence of diffuse p53 overexpression in the SHH subgroup conveyed a difference in overall survival using the log-rank test (*p* < 0.01) however the numbers were too small for statistical power. Three cases had *SUFU* mutations as assessed by gene panel sequencing. One case with an *SUFU* mutation turned out to be unclassifiable using either method. The gene panel screening results are briefly summarized in Fig. 2.

## Discussion

Clinical advances have shifted from risk assessment based on histologic subgroups to molecular subgroups over the last decade and risk stratification is important because the allocation of treatment strategies depends on stratification [18, 20, 21]. The characteristics of four distinct molecular subgroups have been described in multiple studies. The WNT subgroup comprises 10% of all medulloblastomas and is characterized by a good prognosis and peduncular location. The SHH subgroup accounts for approximately 30% of cases and is observed in infants with desmoplastic and nodular histology, intermediate prognosis, and hemispheric location. Group 3 and Group 4 consist of approximately 25% and 35% of cases, respectively, with midline locations, poor and intermediate prognoses, and a lack of characteristic driver mutations [18, 22]. IHC has historically played a major role in molecular subgrouping; however, there are difficulties with the standardization and generalization of this method [12]. The NanoString method has been highlighted due to its reproducibility, consistency, and accuracy [10–12].

Studies of medulloblastoma in the Korean population have been reported but only as histologic subgroups or with IHC-based subgrouping [9, 13]. A study from our institute by Min et al. was published with pathological data of 74 cases from 1999 to 2009 [9]. We have published about tumor-associated macrophage in 45 medulloblastoma cases subgrouped by NanoString assay [17]. Study methods were basically the same with the present study, however IHC was only used for identifying macrophage subtypes not used for molecular subgrouping. The present study is focused on comparison subgrouping results and has clinical significance since it is the most extensive report based on the NanoString assay in the Korean population, and the clinical data are up to date compared to previous papers.

Most of the characteristics of pediatric medulloblastoma in the present study were similar to previously known general characteristics such as age, sex proportion, and rate of metastasis (Table 1) [1]. However, prognosis for the SHH subgroup was worse than known survival data. It was even worse than for Group 4 patients and similar to that of Group 3 patients (Fig. 3). The proportion of large cell/anaplastic histology (30%) seemed to be higher than that in the SHH groups (Fig. 4) [23, 24]. Kaplan–Meier curve showed large cell/anaplastic histology with worse prognosis compared with other histologic subtypes (Fig. 5b). Age and metastasis did not exhibit a significant difference among molecular subgroups. Despite the importance of molecular subgrouping, histologic subgroup especially large cell/anaplastic types should retain their prognostic significance [18]. This finding may implicate that the SHH subgroup may have a more aggressive course in the Korean population, and clinicians should carefully determine whether these patients exhibit large cell/anaplastic features in histological sections. The findings of this study may also indicate that ethnic differences in clinical characteristics exist in medulloblastoma [14, 15]. These ethnic disparities have also been observed in other embryonal tumors such as Wilm’s tumor and neuroblastoma endorsing the results of our study [25, 26].

Further analysis of poor prognostic factors such as *MYC/MYCN* amplification and *TP53* mutation was performed. *MYC/MYCN* amplification was present in 14.8% of observed cases and was correlated with poor overall survival (Fig. 5a). The *MYCN* amplification was not exceptionally high in SHH subgroup (29.4%, five out of 17 patients) compared with other literatures [24, 27]. The presence of *MYC/MYCN* amplification show correlation with anaplastic histology which was also observed in our study [28], however in SHH subgroup there was only one patient who both showed *MYCN* amplification and large cell/anaplastic histology. Both *MYCN* amplification and large cell/anaplastic histology are known as independent risk modifier in SHH subgroup [29] and considering the notable proportion of large cell/anaplastic histology it could be more responsible for aggressive course of SHH subgroup. Statistical analysis is limited due to small number of cases with each risk factors, therefore further analysis of Korean population data is necessary. The p53 IHC does not precisely reflect the presence of *TP53* mutation. However, overexpression of p53 in greater than 50% of cells exhibited a correlation with *TP53* mutation [30]. In our data, there were two cases with p53 overexpression in more than 90% of cells that were stained and considering that these patients had aggressive clinical courses (overall survival approximately one year), they may have had *TP53* mutations. The accurate proportion of *TP53* mutations in our study is difficult to know with certainty, because of limited number of analysis cases. but assuming that those two cases out of eight (25%) in the SHH group were *TP53* mutant, the proportion of *TP53* mutations in our cohort does not seem significantly high compared figures in the literature [24, 27]. Further evaluation of *TP53* status in our institute is necessary for risk stratification and addition of *TP53* to the NanoString codeset might be an option [31].

Using the NanoString assay, we were able to distinguish molecular subgroups in 90% of patients, excluding 10 unclassifiable cases. In these 10 cases, the assay had proceeded normally after RNA integrity verification so the amount of tissue or the quality of RNA preparations are less likely to be problematic. Clinical characteristics such as age, sex, presence of metastasis, histology, and prognosis of the unclassifiable cases displayed close resemblance to the non-WNT/non-SHH subgroups with no driver mutation reported in previous literature in which the four subgroups were further divided by DNA methylation profiles [24]. Currently, the most accurate tests for medulloblastoma subgrouping are RNA sequencing or methylation array [32]. However, these sophisticated methods are more expensive and difficult to apply in individual clinical units, so there is an ongoing need for more simplified and easily applicable methods [33]. Although the NanoString is a simple assay using a limited gene expression set, it has resulted in accurate (~ 98%), highly sensitive, rapid, and reproducible results, exhibiting high concordance with methylation profiling [10, 12]. The cost of the NanoString assay is much less expensive than microRNA array or RNA sequencing [12, 34]. Therefore, the NanoString assay represents a good alternative, feasible method for molecular subtyping.

Compared to IHC, the NanoString assay has comparative cost-effectiveness [11]. The IHC method has unavoidable limitations due to preparation of the specimen, antibody inconsistency, and interpreter-dependent results. Determining the WNT subgroup using beta-catenin and DKK1 and the SHH subgroup using SFRP1 and GAB1 exhibited relatively high sensitivity. In contrast, subgrouping of Groups 3 and 4 using NPR3 and KCNA1 seemed to have disappointing diagnostic value [10]. Differentiation between Group 3 and Group 4 has been difficult and ambiguous since unlike the WNT and SHH groups there were no distinct driver mutations in these subgroups. In the 2016 and 2021 WHO classifications, they announced the classification of Group 3 and Group 4 as a single group called the non-WNT/non-SHH group largely due to the difficulties of differentiating between the two groups without cumbersome, expensive analyses [5]. Group 3 and Group 4 are subdivided into several minor subgroups, and this simple dichotomy may not reflect the true heterogeneity of non-WNT/non-SHH medulloblastomas [24, 27]. However, differences in age of onset, metastatic potential, and prognosis still exist between the two subgroups which was also observed in our study [29]. The strong inconsistent result especially for Group 3 is noticeable. The low subgroup specificity of NPR3 may attribute to our result and therefore NPR3 alone may not be used as marker for Group 3 [12]. When NanoString assay is not available, addition of FISH for *MYC/MYCN* amplification may help distinguishing Group 3 as Kaur et al. have suggested [11]. We were also able to identify five more Group 3 patients by adding results of *MYC/MYCN* amplification.

There are some limitations to this study. Because this was a single institution study, there may be selection bias and it is difficult to represent all Korean pediatric patients. Another limitation is that the result of the NanoString assay was not confirmed using more accurate methods such as RNA sequencing and methylation assays. Our assessment of the accuracy of the NanoString assay depends on previously reported literature [10, 11].

## Conclusion

With the NanoString assay of medulloblastoma we observed an aggressive subgroup among the SHH subgroup which may be explained by the higher prevalence of large cell/anaplastic histology that was observed. Risk stratification and treatment strategies focusing the SHH subgroup should be performed with more attention in Korean pediatric population. IHC is feasible for molecular subgrouping especially for WNT and SHH subgroups, but distinguishing Group 3 from Group 4 may not be possible due to a lack of accuracy. The NanoString assay may represent a good alternative method for practical use in the clinical field.

## Supplementary Information

**Additional file 1.**

## Data Availability

The dataset supporting the conclusions of this study is available on request from the corresponding author. The data are not publicly available due to privacy or ethical restrictions.

## Abbreviations

IHC: Immunohistochemistry
WNT: Wingless signaling-activated
SHH: Sonic-hedgehog signaling-activated
FISH: Fluorescence in situ hybridization
