# Supplementary material for: Molecular subgrouping of medulloblastoma in pediatric population using the NanoString assay and comparison with immunohistochemistry methods
Source: BMC Cancer. 2022 Nov 28;22:1221. doi: 10.1186/s12885-022-10328-6 (PMC9703680; doi:10.1186/s12885-022-10328-6)
Supplement: Supplementary file 1 — Additional file 1. [file 12885_2022_10328_MOESM1_ESM.docx]

Supplementary Table 1. NanoString codeset (PMID: 22057785)

| **Gene name** | **Accession** | **Design Remarks** |
| --- | --- | --- |
| TNC | NM_002160.1 | WNT |
| WIF1 | NM_007191.2 | WNT |
| DKK2 | NM_014421.2 | WNT |
| GAD1 | NM_000817.2 | WNT |
| EMX2 | NM_004098.3 | WNT |
| SFRP1 | NM_003012.3 | SHH |
| ATOH1 | NM_005172.1 | SHH |
| HHIP | NM_022475.1 | SHH |
| PDLIM3 | NM_014476.3 | SHH |
| EYA1 | NM_172059.2 | SHH |
| GABRA5 | NM_000810.2 | G3 |
| NPR3 | NM_000908.2 | G3 |
| NRL | NM_006177.3 | G3 |
| MAB21L2 | NM_006439.4 | G3 |
| IMPG2 | NM_016247.2 | G3 |
| EGFL11 | NM_198283.1 | G3 |
| OAS1 | NM_016816.2 | G4 |
| EOMES | NM_005442.2 | G4 |
| KCNA1 | NM_000217.2 | G4 |
| UNC5D | NM_080872.2 | G4 |
| KHDRBS2 | NM_152688.2 | G4 |
| RBM24 | NM_153020.2 | G4 |
| LDHA | NM_005566.1 | housekeeping |
| GAPDH | NM_002046.3 | housekeeping |
| ACTB | NM_001101.2 | housekeeping |

Supplementary Table 2. Basic Characteristics

| Clinical Features | Number of Cases (%) or value |
| --- | --- |
| Gender |  |
| M | 63 (62.4%) |
| F | 38 (37.6%) |
| Age, years | 7.7 ± 4.7 |
| ≤3 | 23 (22.8%) |
| 3-18 | 78 (77.2%) |
| Metastasis at diagnosis |  |
| Negative | 63 (62.4%) |
| Positive | 38 (37.6%) |
| Tumor location |  |
| Midline | 82 (81.2%) |
| Hemispheric | 13 (12.9%) |
| Peduncle | 6 (5.9%) |
| Extent of surgery |  |
| Gross total resection | 49 (48.5%) |
| Near total resection | 42 (41.6%) |
| Subtotal resection | 10 (9.9%) |
| Histology |  |
| Classic | 61 (60.4%) |
| MBEN | 17 (16.8%) |
| DN | 9 (8.9%) |
| LCA | 14 (13.9%) |
| Subgroup (by IHC method) |  |
| WNT | 13 (12.9%) |
| SHH | 21 (20.8%) |
| Group 3 | 21 (20.8%) |
| Group 4 | 23 (22.8%) |
| Unclassifiable | 7 (6.9%) |
| Insufficient data | 16 (15.8%) |
| Subgroup (by NanoString method) |  |
| WNT | 14 (13.9%) |
| SHH | 20 (19.8%) |
| Group 3 | 18 (17.8%) |
| Group 4 | 39 (38.6%) |
| Unclassifiable | 10 (9.9%) |

Abbreviations: MBEN, Medulloblastoma with extensive nodularity; DN, Desmoplastic/nodular; LCA, Large cell/anaplastic; WNT, Wingless signaling-activated; SHH, Sonic-hedgehog signaling-activated.
